# The Biological Implication of Semicarbazide-Sensitive Amine Oxidase (SSAO) Upregulation in Rat Systemic Inflammatory Response under Simulated Aerospace Environment

**DOI:** 10.3390/ijms24043666

**Published:** 2023-02-11

**Authors:** Liben Yan, Chunli Sun, Yaxi Zhang, Peng Zhang, Yu Chen, Yifan Deng, Tianyi Er, Yulin Deng, Zhimin Wang, Hong Ma

**Affiliations:** 1 School of Life Science, Beijing Institute of Technology, Beijing 100081, China; 2 Advanced Research Institute of Multidisciplinary Science, Beijing Institute of Technology, Beijing 100081, China

**Keywords:** simulated aerospace environment, semicarbazide-sensitive amine oxidase, inflammatory response, myocardial damage

## Abstract

The progress of space science and technology has ushered in a new era for humanity’s exploration of outer space. Recent studies have indicated that the aerospace special environment including microgravity and space radiation poses a significant risk to the health of astronauts, which involves multiple pathophysiological effects on the human body as well on tissues and organs. It has been an important research topic to study the molecular mechanism of body damage and further explore countermeasures against the physiological and pathological changes caused by the space environment. In this study, we used the rat model to study the biological effects of the tissue damage and related molecular pathway under either simulated microgravity or heavy ion radiation or combined stimulation. Our study disclosed that ureaplasma-sensitive amino oxidase (SSAO) upregulation is closely related to the systematic inflammatory response (IL-6, TNF-α) in rats under a simulated aerospace environment. In particular, the space environment leads to significant changes in the level of inflammatory genes in heart tissues, thus altering the expression and activity of SSAO and causing inflammatory responses. The detailed molecular mechanisms have been further validated in the genetic engineering cell line model. Overall, this work clearly shows the biological implication of SSAO upregulation in microgravity and radiation-mediated inflammatory response, providing a scientific basis or potential target for further in-depth investigation of the pathological damage and protection strategy under a space environment.

## 1. Introduction

During human evolution, disease is often accompanied by the existence of pain and death. In today’s world, heart disease, diabetes, neurodegenerative disease, tumor, and other major diseases are serious threats to human health [1,2,3,4]. Inflammation has been shown to be an important mechanism for the progression of most common diseases [5]. Among the various important biological indicators, semicarbazide sensitive amino oxidase (SSAO), expressed in most tissues and organs [6,7], has been shown to have a close relationship with these inflammatory responses [8,9,10,11]. Some studies have revealed that the sequence of vascular adhesion protein-1 (VAP-1) is consistent with the cDNA of SSAO [12], showing the same distribution and mode of action. Therefore, many previous works have focused on the functional identification of VAP-1 with SSAO [13]. For instance, soluble VAP-1 is upregulated and released in certain inflammatory disorders such as diabetes [14,15] and hepatitis [16]. In humans, serum VAP-1 is associated with atherosclerosis in carotid arteries [17,18] and the incidence of some major cardiovascular events [19,20]. Moreover, serum VAP-1 can predict cardiovascular mortality accompanied with type 2 diabetes [21,22] and the general population [23]. Yu et al. found that in SSAO-overexpressing transgenic mice, the activity of SSAO and the number of neutrophils in bronchoalveolar lavage fluid was significantly increased, and the levels of granulocyte colony-stimulating factor, IL-6 and TNF-α were also upregulated [24]. Zhang et al. demonstrated that the activity of SSAO in blood changed regularly during the onset of acute pneumonia, and that the increase or decrease in its activity could affect the inflammatory reaction process [25]. In vitro experiments have also indicated that inflammatory factors such as TNF-α, IL-1β, and IL-4 can stimulate the expression level of SSAO/VAP-1.

With the continuous advancement in the major scientific and technological projects of manned spaceflight, mankind has turned the space program to planetary exploration, aiming to send astronauts to Mars and the Moon [26,27,28]. However, due to the existence of complex factors such as microgravity, radiation, magnetic field, and circadian rhythm change in the space environment, various physiological indicators of astronauts will be affected to a certain extent during their stay in orbit [29,30,31,32,33,34], which may interfere with the normal progress of space missions [35,36,37,38]. Epidemiological results show that exposure to radiation at a dose less than 0.5 Gy can significantly increase the risk of radiation-induced cardiovascular disease [39]. Studies have shown that microgravity can cause microtubule loss, a decrease in mitochondrial number, and a decrease in the cell cross-sectional area in cardiomyocytes [40,41,42]. Zheng et al. found that simulated microgravity could cause changes in the gene expression levels in rat cardiomyocytes, and the expression levels of protective factors were reduced, while some inflammatory factors were significantly upregulated, indicating that simulated microgravity would have a negative impact on cardiomyocytes [43,44]. However, the mechanisms related to SSAO and cardiovascular injury-associated inflammatory response in the space environment are still unknown.

In this work, we investigated the biological effects of the tissue damage and related molecular pathway in a simulated space environment exposed rat model and genetic engineered cell lines. By using histomorphology, biochemical analysis, and Western blot verification, the correlation of SSAO on the inflammatory response in damaged tissues under simulated microgravity and heavy ion radiation was studied. At the same time, the molecular mechanisms behind the pathological phenotype changes were profiled in detail. Our study disclosed that SSAO upregulation is closely related to the systematic inflammatory response (IL-6, TNF-α) in rats under a simulated aerospace environment. In particular, the simulated space environment leads to significant changes in the level of inflammatory genes in the heart tissues and systemic circulation, thus causing different inflammatory responses among different tissues, which might be related to altering the expression and activity of SSAO. In summary, this study provides a scientific basis for further in-depth investigations of space environment-caused pathological damage, benefiting the development of an effective biomedical protecting strategy for manned space exploration.

## 2. Results

### 2.1. Simulated Space Environment Induced Changes of Physiological Characteristics and Inflammatory Factors of Serum in Rats

**Figure 1:**
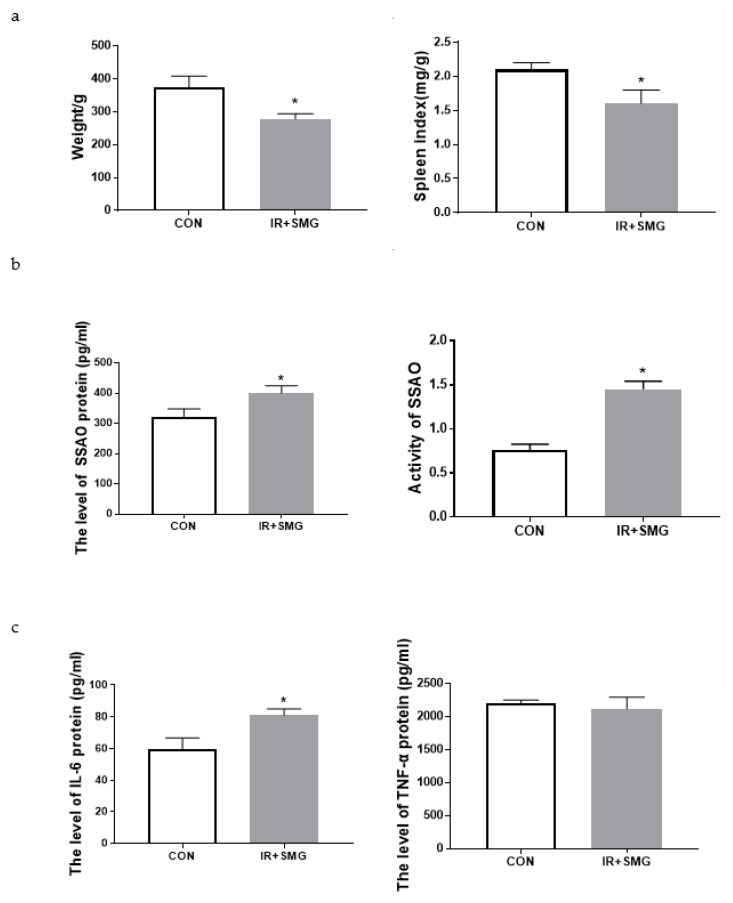
Effects of the simulated space environment on the rat physiological changes. (**a**) Body weight and spleen index of rats in different groups. (**b**) The expression of the SSAO protein and its activity in the serum of rats in different groups. (**c**) The expression of inflammatory factors in the serum of rats in different groups. Data are expressed as the mean ± SD (*n* = 12). Compared with the CON group, * *p* < 0.05.

We first evaluated the physiological and pathological indications of the spatial environment rat model. The results of the rat body weight can be seen in Figure 1a. Compared with the control group (CON), the body weight of rats at 21 days after the treatment of the simulated space environment (IR + SMG) decreased significantly. The spleen index of rats, representing the immune ability, was significantly downregulated in the treated group compared with that of the control group (Figure 1a). Further analysis showed that the simulated space conditions significantly increased the expression level of SSAO protein and SSAO enzyme activity in the rat serum (Figure 1b). Moreover, compared with the CON group, the protein expressions of IL-6 in the serum of rats of the IR + SMG group were significantly increased (Figure 1c). Taken together, the simulated space environment can cause physiological changes and produce inflammatory factors, and the change in the SSAO level is consistent with the change trend in IL-6, indicating that serum SSAO is highly correlated with systemic inflammatory response.

### 2.2. Changes of Different Tissues in Rats Induced by Simulated Space Environment

**Figure 2:**
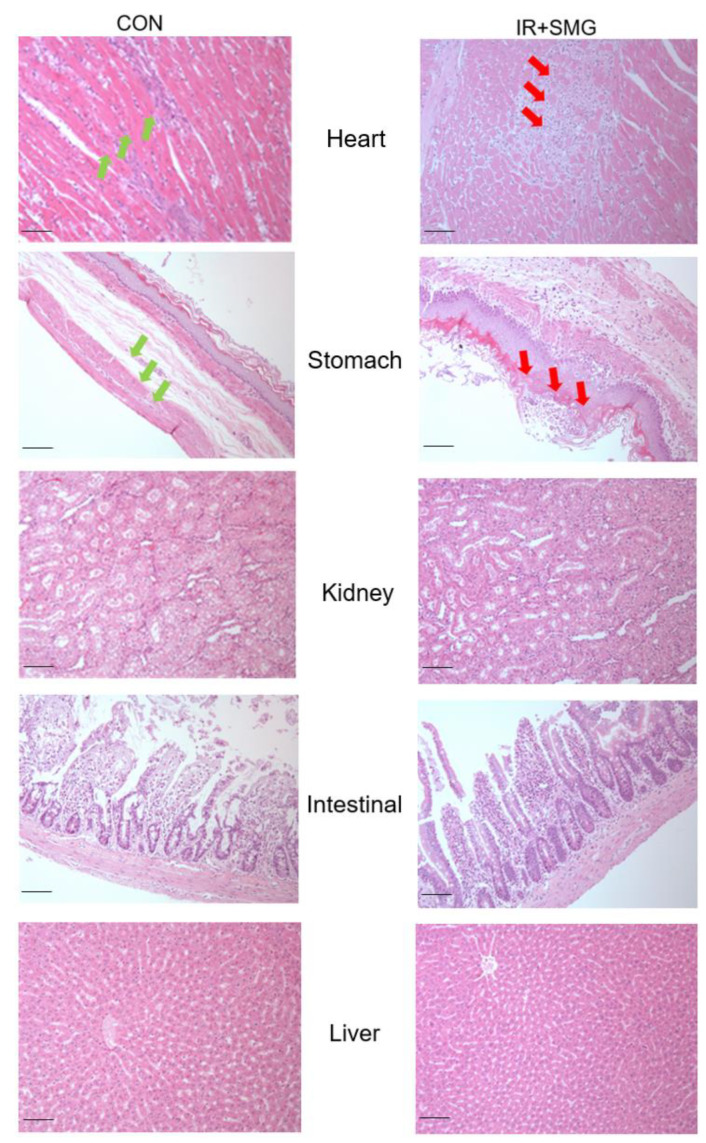
H&E staining of the rat tissues in each group. Inflammatory and normal cells are marked with red and green arrows, respectively; the length of scale bar was 100 μm.

In order to further evaluate the effect of systemic inflammatory response on different tissues caused by the simulated space environment, the immunohistochemistry staining method was used to evaluate the pathological characteristics of tissues from the rats in our experimental model. The results of the hematoxylin and eosin (H&E) staining are shown in Figure 2. Compared with the control group (CON), the heart tissue and stomach tissue of rats in the simulated space environment showed obvious inflammatory infiltration (red arrow), and the gastric tissue showed glandular gastric mucosa autolysis and the heart tissue showed focal fibrous hyperplasia. However, there was no significant inflammatory damage in the kidney, intestine, and liver tissue of the rats in the simulated space environment compared with the control group. Based on these results, we can conclude that different organs and tissues might have a different sensitivity to the space environment, which may be related to the characteristics of different tissues. We found that the most sensitive heart tissue showed clear pathological changes accompanied by inflammation in the simulated space environment, while the least sensitive kidney tissue did not show any changes.

### 2.3. The Biological Effects of Space Environment on Rat Heart and Kidney Tissue Were Simulated

**Figure 3:**
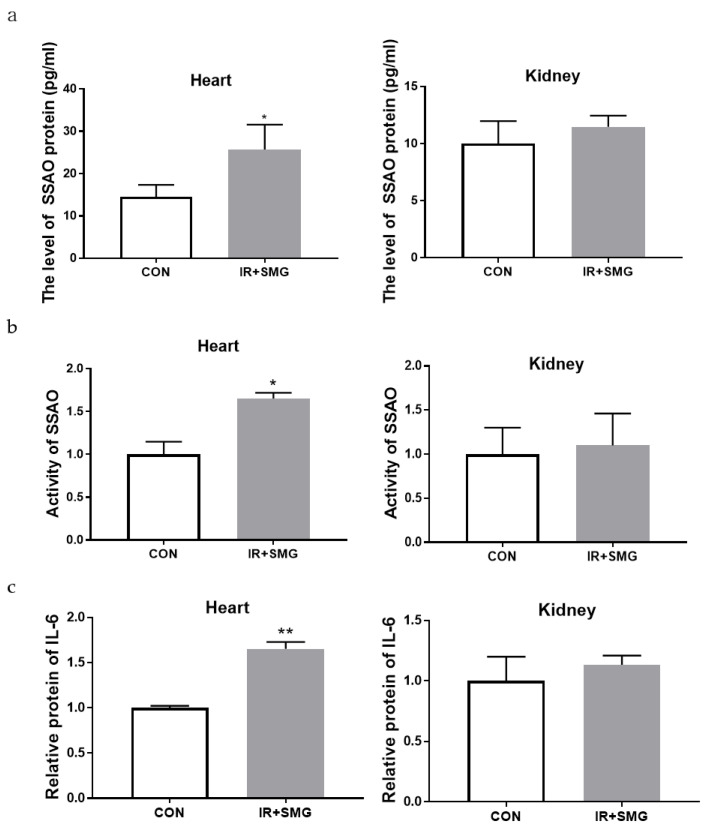
The effects of the simulated spatial environment on cardiac and renal tissues in rats. (**a**) The expressions of the SSAO protein in the heart and kidney of rats in different groups were detected by ELISA. (**b**) The SSAO activity in the heart and kidney of rats in different groups. (**c**) The expression of IL-6 in the heart and kidney of rats in different groups. Data are expressed as the mean ± SD (*n* = 12). Compared with the CON group, * *p* < 0.05 and ** *p* < 0.01.

Based on the correlation between SSAO and tissue inflammatory damage, we evaluated the expression level and enzyme activity of SSAO in different tissues. The expression of all SSAO proteins in the heart tissue was also significantly increased in the treatment group compared with the CON group, but there was no change in the renal tissue (Figure 3a). Compared with the control group (CON), the activity of the SSAO enzyme in the simulated space environment was significantly increased in the heart tissue, while there was no significant change in the kidney tissue (Figure 3b). The simulated space environment also altered the level of expressed IL-6 in the heart tissue, but had no appreciable effect on the kidney tissue (Figure 3c). Overall, SSAO is highly correlated with pathological injury and the inflammatory response of heart tissue in simulated space environments, suggesting that SSAO and IL-6 have a synergistic effect and that they may jointly mediate the occurrence of inflammatory response changes in different tissues.

### 2.4. Bioactivity of Human Cardiomyocyte AC16 and Human Embryonic Kidney HEK293 Cells Overexpressed by SSAO

**Figure 4:**
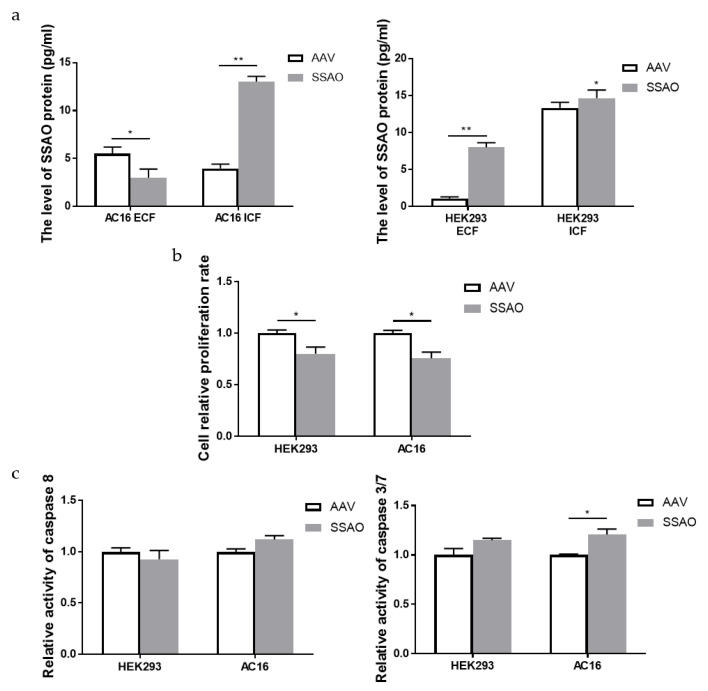
Bioactivity of human cardiomyocytes AC16 and human embryonic kidney cells HEK293 overexpressed with SSAO (ECF: extracellular fluid and ICF: intracellular fluid). (**a**) Analysis of SSAO expression after transfection of recombinant AAVs in two cell lines. (**b**) Survival rate of two types of cells after SSAO overexpression. (**c**) Assessment of the enzyme activities of Caspase8 (left lane) and Caspase 3/7 (right lane) were evaluated after SSAO overexpression in different cells (mean ± standard deviation). * *p* < 0.05, ** *p* < 0.01 compared to the relevant AAV changes.

In order to further detect the correlation between SSAO and tissue cell damage, we constructed the system of SSAO overexpression and detected intracellular and extracellular SSAO from different tissue sources after the upregulation of SSAO. We made the recombinant AAV vector overexpress the SSAO in different target cells including human cardiomyocyte AC16 and human embryonic kidney HEK293 cells (the construction and viability of recombinant AAVs is shown in the Supplementary Figures S1–S4). In Figure 4a, we can see the successful expression of SSAO mediated by recombinant AAVs, but there were some obvious differences between the cardiomyocyte and embryonic kidney cells. The expression of SSAO in the AC16 cells was mainly in the intracellular part and HEK293 in the extracellular media. The survival rates of the two SSAO overexpression cells are shown in Figure 4b. Compared with the no-load group (AAV), the survival rates of the two SSAO overexpression cells were significantly decreased. The activity of two factors related to apoptosis (Caspase 3/7 and Caspase 8) showed that the activities of Caspase 8 had no change in the AC16 and HEK293 cells, with SSAO overexpression compared with the no-load group, and Caspase 3/7 was significantly upregulated only in the AC16 cells (Figure 4c). In conclusion, SSAO overexpression mediated by virus transfection can inhibit cell proliferation and promote the apoptosis of AC16 cells with Caspase 3/7 activation, which might be related to the intracellular location of SSAO.

### 2.5. Effect of SSAO Overexpression on Inflammatory Response of Human Cardiomyocytes AC16 and Human Embryonic Kidney Cells Hek293

**Figure 5:**
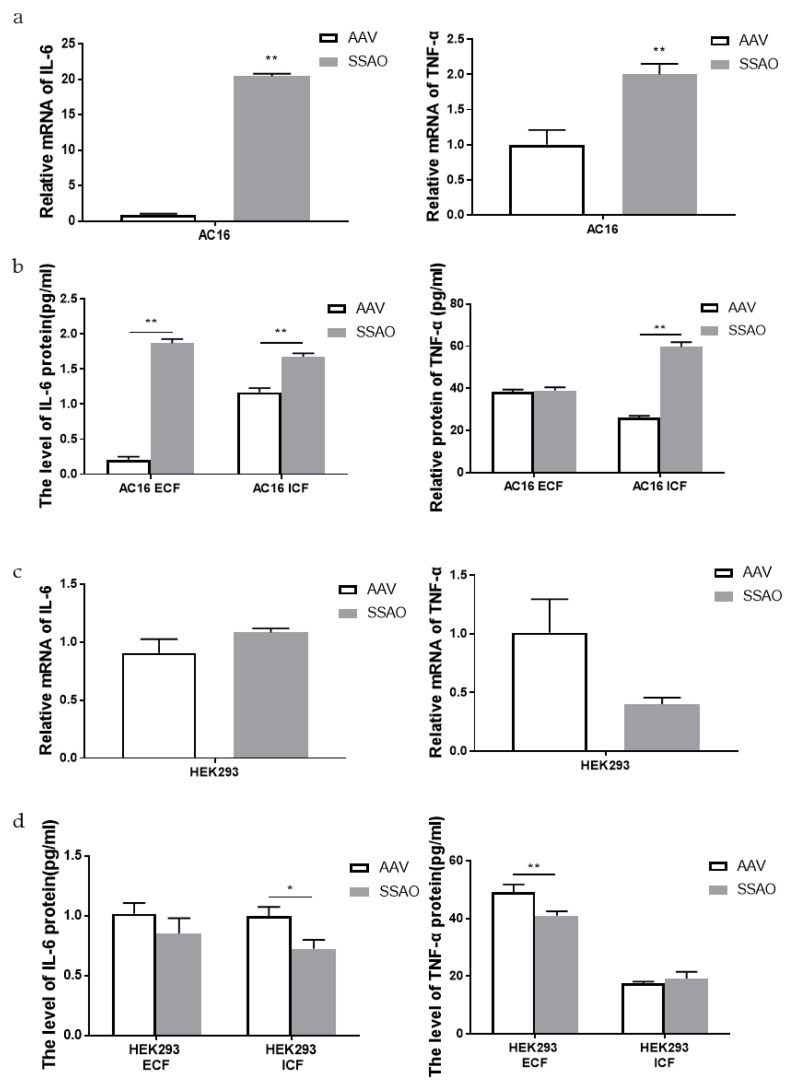
Effect of SSAO overexpression on the inflammatory response of human cardiomyocytes AC16 and human embryonic kidney cells Hek293. (**a**) Transcription levels of IL-6 and TNF-α in the AC16 cell lines after SSAO overexpression. (**b**) Analysis of IL-6 and TNF-α expression in the extracellular and intracellular fluid of the AC16 cell lines after SSAO overexpression. (**c**) Transcription levels of IL-6 and TNF-α in the HEK293 cell lines after SSAO overexpression. (**d**) Analysis of IL-6 and TNF-α expression in the extracellular and intracellular fluid of the HEK293 cell lines after SSAO overexpression. * *p* < 0.05, ** *p* < 0.01 compared to the relevant AAV.

In order to further explore the mechanism of SSAO induced cell damage, inflammatory factors were detected in different cells with high SSAO expression. We further assayed the inflammatory level of IL-6 and TNF-α after virus transfection and first compared the AC16 cells overexpressing SSAO with the no-load group (AAV). The RT-PCR results showed that the levels of inflammatory cytokines IL-6 and TNF-α were significantly upregulated (Figure 5a). The expression of the IL-6 protein in the extracellular and intracellular fluid was significantly increased, and the TNF-α protein was significantly increased in the intracellular fluid but not changed in the extracellular fluid (Figure 5b). However, we then compared the HEK293 cells overexpressing SSAO with the AAV group. The results showed that the transcriptional levels of inflammatory cytokines IL-6 and TNF-α did not change (Figure 5c). The expression of the IL-6 protein was significantly decreased in the intracellular fluid, but was not changed in the extracellular fluid, while the expression of the TNF-α protein was the opposite to that of the IL-6 protein, which was significantly decreased in the extracellular fluid but not changed in the intracellular fluid (Figure 5d). As a result, the inflammatory response of the HEK293 cells and AC16 cells was different after SSAO overexpression. The levels of inflammatory factors in the HEK293 cells were downregulated or no difference was observed, and both exocytotic and intracellular inflammatory factors were significantly upregulated in the AC16 cells.

## 3. Discussion

In the present study, we determined the damaging effects of the simulated space environment on the body, and confirmed the damaging changes of the simulated space environment on the myocardial inflammatory response. Therefore, we used a combined model of total body irradiation plus tail crane for 21 days to study the changes of the long-term space flight on the body, providing a new angle for exploring the protection and prevention of the body in the space environment. The spatial composite model in this study corresponds to the actual situation of the blood’s redistribution due to the microgravity conditions. As the dynamic center of blood transport in the body, the heart is bound to undergo corresponding changes as the mechanical signals change, and radiation in the environment of long-term space flight will also cause changes in the body [45,46].

**Figure 6:**
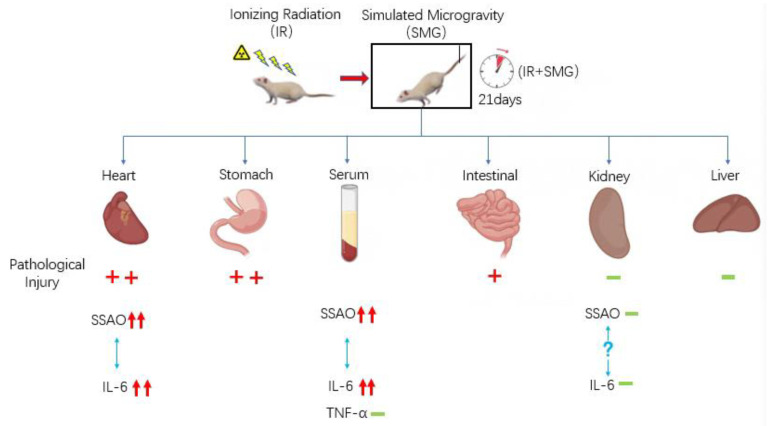
Schematic illustration of the SSAO mediated inflammatory damage in rats under a simulated spatial environment. (+: Degree of damage; −: No obvious damage or change; The degree of protein content is indicated by a red rising arrow.)

Furthermore, the compound condition model had an impact on the growth of the rat body and the spleen, and the serum results showed that the simulated spatial compound condition can cause changes in the serum levels of SSAO and inflammatory factors in rats. Compared with the control group, the content of IL-6 in the serum in the compound condition group significantly increased, while the content of TNF-α did not, and the content and activity of SSAO also obviously increased. There was no obvious correlation between the change trend of SSAO in the serum and TNF-α, while the change in SSAO level was highly consistent with IL-6. This indicates that SSAO in serum also has a great correlation with inflammation and has a synergistic effect with IL-6, which may jointly mediate the occurrence of inflammation. The pathological sections of various organs showed that the sensitivity of different organs to the combined conditions of radiation and microgravity was different, which may be related to the structure, composition, volume, location, and other characteristics of different tissues. Changes in cardiovascular function induced by microgravity in space can further lead to physiological problems during later flight. The damaged cardiovascular system may be more susceptible to the effects of space radiation, and the synergistic effect of both may make cardiovascular damage more pronounced. The pathological section results of this study showed that after the combined action of radiation and microgravity, the inflammatory reaction was more serious than the control group, and the pathological injury of the heart was the most serious, which resulted in myocardial fibrosis. In this study, no significant pathological changes were observed in the kidney tissues of rats in the control group or the combined group of radiation and microgravity, indicating that the kidney was less sensitive to radiation and microgravity under the simulated space conditions. Our preliminary results show that SSAO varies in the heart and kidney, and is associated with the expression of inflammatory cytokines (Figure 6). However, the upstream regulation mechanism of SSAO has not been reported yet. According to the literature research, the mechanisms of action of non-coding RNA include changing the expression of genes and the activity of regulatory proteins [47]. The results of this study showed that the expression and activity of SSAO in the heart and kidney tissues were different. Therefore, we speculated that the increase in SSAO in the heart tissues in this study might be regulated by non-coding RNA. The spatial environment may lead to changes in the level of non-coding RNA in the heart tissue, thus altering the expression and activity of SSAO, and further affecting the changes of the downstream genes. However, the specific roles and effects of microgravity and radiation in the inflammatory damage effects of myocardium seen in the composite model need to be established respectively for research.

Based on the experimental results of the in vivo model, we also explored the effects of SSAO changes on the AC16 cardiomyocytes and HEK293 kidney cells in the in vitro model. In this study, after the infection of the HEK293 cells and AC16 cells under the optimal experimental conditions, the extracellular secretion of the SSAO protein in the HEK293 cells obviously increased while decreased in the AC16 cells, but the intracellular expression level of the SSAO protein in the AC16 cells and HEK293 cells both increased significantly. Compared with the AAV negative control group, the SSAO overexpression cell model was successfully constructed. However, the intracellular and extracellular expression of SSAO were obviously different. The results showed that after SSAO overexpression, the number of HEK293 cells and AC16 cells decreased, indicating that SSAO virus infection had an inhibitory effect on cell proliferation. In HEK293 cells, there were significant differences in the activity of the apoptotic factor, and the level of the apoptotic factor was downregulated while no significant difference was shown. The expression levels of the apoptosis promoter Caspase 8, and apoptotic factor Caspase 3/7 were both significantly increased in the AC16 cells, which indicated that the overexpression of SSAO would cause damage to the AC16 cells. Further study results showed that from the transcription level of inflammatory factors IL-6 and TNF-α greatly increased after the overexpression of SSAO in the AC16 cells, which showed that SSAO significantly upregulated the RNA level of inflammatory factor IL-6 and upregulated the RNA level of inflammatory factor TNF-α. However, after the overexpression of SSAO in the HEK293 cells, the level of inflammatory cytokines IL-6 and TNF-α decreased or had no significant changes. Therefore, it can be considered that the excessive intracellular expression of SSAO will regulate the transcription of inflammatory factors at the transcriptional level, thus causing inflammatory effects and inducing tissue damage. SSAO is a potential target for the treatment of inflammation, and further research is of great significance for the improvement in biological protection strategies for astronauts under long-term flight conditions.

## 4. Materials and Methods

### 4.1. Animal Groups and Treatment

Male 8-week Wistar rats were purchased from Lanzhou Veterinary Research Institute, Chinese Academy of Agricultural Sciences (LVRI, CAAS). All of the experimental rats were humanely treated in accordance with the guidelines for animal care of the Beijing Institute of Technology, and were approved by the Experimental Animal Welfare Ethics Committee of the School of Life Science, Beijing Institute of Technology (SYXK-BIT-20220114001). The experimental animals underwent a week of acclimatization period. The animals were randomly distributed into two groups (*n* = 12): control, radiation, and tail crane combined. Animals in the radiation and tail crane combined group had total-body radiation at the radiation dose of 0.25 Gy/min. The radiation source was ^12^C^6+^ heavy ion rays from Wuwei Heavy Ion Therapy Center in Lanzhou, with the energy of 260 MeV, the energy transfer line density (LET) of 13.9, and the total radiation dose of 0.5 G). Animals in the radiation and tail crane combined group were housed individually in cages at about 30–35 degrees head-down tilt with their tails in such a position that only the forelimbs were free for locomotion and could touch the bottom of the cage [22]. Food and water were available ad libitum. After 21 days, the mice were anesthetized with 3% pentobarbital (50 mg/kg intra-peritoneally, Sigma, Milwaukee, WI, USA). Subsequently, the tissues were fixed with 4% paraformaldehyde solution. Blood samples were collected from the heart into heparinized tubes and centrifuged to obtain serum.

### 4.2. H&E Staining

The rat tissues were fixed with 4% paraformaldehyde (Solarbio, Beijing, China). The fixed tissues were dehydrated with various concentrations of xylene and ethanol and embedded in paraffin [48]. The 5 µm sections were sliced and stained with H&E. Each tissue section was scanned using a Nano Zoomer S210 microscopic resolution scanner (Hamamatsu Corporation, Hamamatsu, Japan).

### 4.3. RT-PCR

Total RNA from each tissue and cell was isolated using the Trizol reagent (Invitrogen, Carlsbad, CA, USA). RNA from different samples was reverse transcribed to cDNA for gene expression detection by reverse transcription polymerase chain reaction (RT-PCR). For reverse transcription, 2 μg RNA was added in the EasyScript^®^ First-Strand cDNA Synthesis SuperMix (Transgen Biotech, Beijing, China) and then kept for 30 min at 37 °C. GAPDH levels were used for normalization and non-reversed transcribed RNA was used to correct for the presence of genomic DNA. After adding PCR SuperMix (Transgen Biotech, Beijing, China), cDNA, and primers, the following procedure was performed. Thirty cycles of PCR were conducted with a pre-denaturation at 94 °C for 5 min, and 30 cycles of PCR (denaturation at 94 °C for 40 s, annealing at 54 °C for 30 s, extension at 72 °C for 40 s). A final extension was carried out at 72 °C for 10 min, and the samples were then kept at 4 °C. Primer pairs for four genes are shown, GAPDH forward: 5′-GGCCAACGCATGTTCTTCAG-3′, Reverse: 5′-TTTGTGATGCGTGTGTAGCG-3′; for SSAO, Forward: 5′-AACCAGAATGA CCCGTGGAC-3′, Reverse: 5′-CCCACCCGTGAGAAAGTGAA-3′; for IL-6, Forward: 5′-AACAAGAGGTGAGTGCGTCC-3’, Reverse: 5′-CTGCTACCCTGAGATGCTGG-3′ and for TNF-α, Forward: 5′-TAYCCGTCCAACCTCAGCATC-3′, Reverse: 5′-GGAACAGGGAAGAAGCAAGAGA-3′.

### 4.4. ELISA

The homogenates of rat tissue were prepared and centrifuged. Supernatants were collected for the biochemical assays. The contents of TNF-α and IL-6 in the rat tissue, serum, and cells were determined with an ELISA Kit (Invitrogen, Carlsbad, CA, USA) according to the manufacturer’s instructions.

### 4.5. Packaging and Extraction of Adeno-Associated Virus

The human adeno-associated virus pCA-CAG-P2A-AOC3 was constructed and packaged into virus particles to transfect the passaged AC16 cells and HEK293 cells to obtain a high AOC3 gene expressing AC16 cells and HEK293 cells. The 5 × 10^5^ AC16 cells and HEK293 cells were seeded in a 3.5 cm-dish and cultured at 37 °C. When the confluent cells were about 70–80%, the medium was removed and replaced with fresh medium (without antibiotics) and cultured at 37 °C. The plasmid was transfected with Lipofectamine 2000 (Sigma, Milwaukee, WI, USA) and incubated at 37 °C. After 4 h of incubation, the cells were cultured in fresh complete DMEM medium containing 10% of FBS, 1% of antibiotics, and 1% of GlutaMAX. After 36–48 h of transfection, the cells were digested with trypsin and cultured in a 10 cm-dish. After that, fresh culture medium was changed every 2–3 days. Obvious virus spots appeared in 5–7 days, and many cells disintegrated and fell off. The harvested supernatant of the cell culture and cell fragments were thawed twice to lyse the cells. The virus solution was repeatedly frozen, thawed, and centrifuged at 3000 rpm for 15 min to remove the cell debris. The virus crude extract was separately packed in a 1.5 mL centrifuge tube and stored at −80 °C.

### 4.6. SSAO Activity Analysis

SSAO activity was determined as a result of the turnover of hydrogen peroxide, which was measured by the Amplex Red Monoamine Oxidase Assay Kit (Invitrogen, Beijing, China). Briefly, 100 g of protein was incubated at room temperature with clorgyline 1 M (monoamine oxidase-A inhibitor), pargyline 3 M (monoamine oxidase-B inhibitor), benzylamine 2 mM (substrate of SSAO), Amplex Red reagent 100 M, and horseradish peroxidase 1 U/mL, with or without semicarbazide 1 mM. Absorbance at 570 nm was measured every 5 min for 30 min. A standard curve was plotted using different solutions with known hydrogen peroxide concentrations. The production rate of hydrogen peroxide was calculated and expressed as [H_2_O_2_]/min/g protein. SSAO activity was determined by the difference between the production rates of hydrogen peroxide with and without semicarbazide.

### 4.7. Cells and Culture

The human cardiac myocytes AC16 and the human embryonic kidney cell line HEK293 were obtained from the Cell Center of Peking Union Medical College (Beijing, China). DMEM or 1640 supplemented with 10% heat-inactivated fetal bovine serum (Gibco, Carlsbad, CA, USA), 100 units/mL penicillin, and 100 μg/mL streptomycin (Beijing Solarbio Science & Technology Co., Beijing, China) was used for cell culture. The culture was maintained at 37 °C in a humidified incubator containing 5% CO_2_.

### 4.8. Caspase Activity Assays

Caspase 3/7 and Caspase 8 activation were performed using the ApoOne Homogenous Caspase-3/7 Assay and Caspase-8 Assay (Promega, Madison, WI, USA), respectively, according to the manufacturer’s instructions. Briefly, 1 × 10^4^ cells (treated with or without irradiation) were collected at different time points (24, 48, and 72 h) and lysed in the provided reagent, which contained the profluorescent substrate with an optimized bifunctional cell lysis/activity buffer for Caspase 3/7 or Caspase 8, and then incubated at room temperature for 2 h before being read in a fluorometer at 484/530 nm. The relative caspase activity was given to evaluate the apoptosis level.

### 4.9. Statistical Analysis

All experiments were performed in triplicate each time. The results were shown as the mean values ± standard deviation (SD). The error bars represent the SD of at least three replicates in three independent experiments. Statistical significance was evaluated by the Student’s *t*-test and ANOVA assay. Differences were considered statistically significant at *p* < 0.05.

## Data Availability

Not applicable.

## Supplementary Materials

The following supporting information can be downloaded at: https://www.mdpi.com/article/10.3390/ijms24043666/s1.
