# Supplementary material for: The Biological Implication of Semicarbazide-Sensitive Amine Oxidase (SSAO) Upregulation in Rat Systemic Inflammatory Response under Simulated Aerospace Environment
Source: Int J Mol Sci. 2023 Feb 11;24(4):3666. doi: 10.3390/ijms24043666 (PMC9961990; doi:10.3390/ijms24043666)
Supplement: Supplementary file 1 [file ijms-24-03666-s001.zip › ijms-2100997-supplementary.pdf]

Supplementary Materials

The biological implication of semicarbazide-sensitive amine oxidase (SSAO) upregulation in rat systemic inflammatory response under simulated aerospace environment

Liben Yan [1#], Chunli Sun [1#], Yaxi Zhang [1], Peng Zhang [1], Yu Chen [1,3], Yifan Deng [1], Tianyi Er [1], Yulin Deng [1], Zhimin Wang [1,2*] and Hong Ma [1*]

[1] School of Life Science, Beijing Institute of Technology, Beijing, 100081, China;

[2] Advanced Research Institute of Multidisciplinary Science, Beijing Institute of Technology, Beijing 100081, China.

[3] Aerospace Medical Center, Aerospace Center Hospital，Beijing 100049，China

[#] Liben Yan and Chunli Sun contributed equally to this work.

[*] Correspondence: zmwang@bit.edu.cn; 04656@bit.edu.cn; Tel.: +86-010-68915996

(a)
(c)
(b)

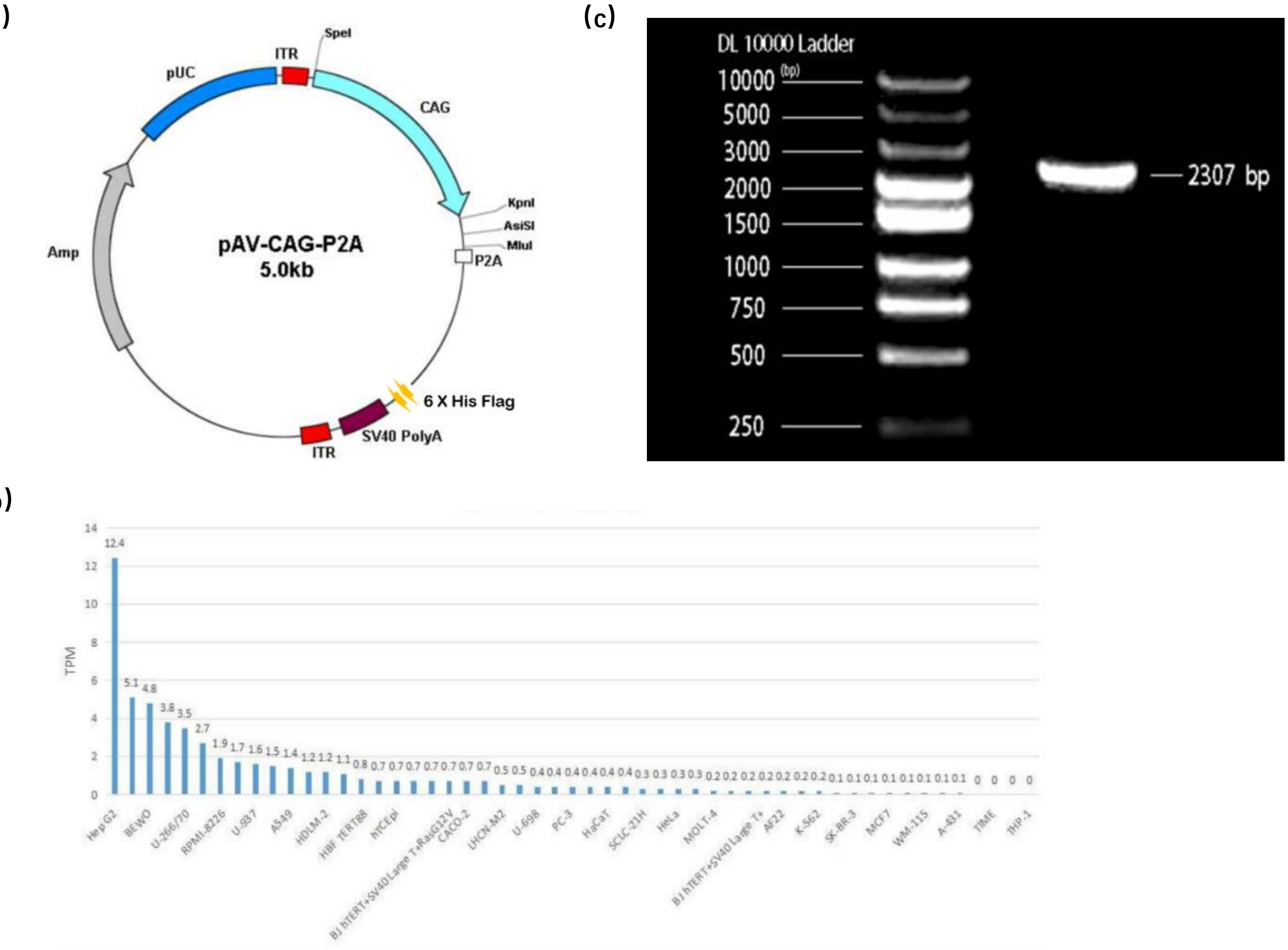

Supplementary Figure S1. (a) Adeno-associated Virus (pAV-CAG-P2A) for AOC3 overexpression. (b)AOC3 gene RNA expression in cells. (c) Identification of SSAO coding genes（AOC3）by 1% gel electrophoresis.

Human hepatoma cell HepG2 (purchased from Cell Center of Institute of Basic Medicine, Chinese Academy of Medical Sciences). Human hepatocellular carcinoma HepG2AOC3 has the highest content. In this study, total RNA was extracted from hepatoma cell line Hep-G2 and cDNA was obtained by reverse transcription. The monoamine oxidase SSAO coding gene (AOC3) was validated and recovered by PCR amplification and agarose gel electrophoresis. The gel electrophoresis was verified by PCR, and the AOC3 connected fragment was obtained successfully.

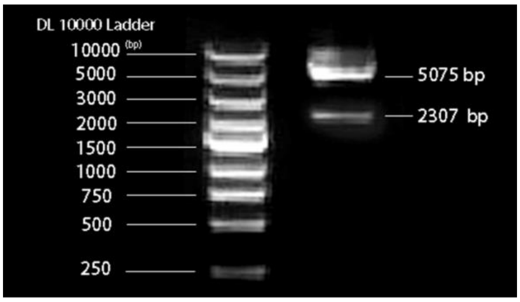

Supplementary Figure S2. Double enzyme digestion of plasmid of recombinant virus vector.

According to enzyme digestion identification, two restriction endonuclease enzymes, AsiSI and MluI, were used to perform enzyme digestion on the recombinant plasmid, and two sequences of different sizes could be obtained, the sizes of which were about 2.3kb and 5kb respectively, corresponding to the length of AOC3 genome and the size of pcDNA4 plasmid. This indicated that AOC3 gene had been successfully integrated into the plasmid, and the recombinant plasmid was well constructed.

```
Alignment of DNAMANpAV-CAG-P2A-SSAO.seq(upper line) and DNAMANssao.seq(lower line)
Identity=100.00%(2328/2328)  Gap=0.00%(0/2328)
-----------------

1      GCGATCGCCACCATGAACCAGAAGACAATCCTCGTGCTCCTCATTCTGGCCGTCATCACC
       ||||||||||||||||||||||||||||||||||||||||||||||||||||||||||||
1      GCGATCGCCACCATGAACCAGAAGACAATCCTCGTGCTCCTCATTCTGGCCGTCATCACC

61     ATCTTTGCCTTGGTTTGTGTCCTGCTGGTGGGCAGGGGTGGAGATGGGGGTGAACCCAGC
       ||||||||||||||||||||||||||||||||||||||||||||||||||||||||||||
61     ATCTTTGCCTTGGTTTGTGTCCTGCTGGTGGGCAGGGGTGGAGATGGGGGTGAACCCAGC

121    CAGCTTCCCCATTGCCCCTCTGTATCTCCCAGTGCCCAGCCTTGGACACACCCTGGCCAG
       ||||||||||||||||||||||||||||||||||||||||||||||||||||||||||||
121    CAGCTTCCCCATTGCCCCTCTGTATCTCCCAGTGCCCAGCCTTGGACACACCCTGGCCAG

181    AGCCAGCTGTTTGCAGACCTGAGCCGAGAGGAGCTGACGGCTGTGATGCGCTTTCTGACC
       ||||||||||||||||||||||||||||||||||||||||||||||||||||||||||||
181    AGCCAGCTGTTTGCAGACCTGAGCCGAGAGGAGCTGACGGCTGTGATGCGCTTTCTGACC

241    CAGCGGCTGGGGCCAGGGCTGGTGGATGCAGCCCAGGCCCGGCCCTCGGACAACTGTGTC
       ||||||||||||||||||||||||||||||||||||||||||||||||||||||||||||
241    CAGCGGCTGGGGCCAGGGCTGGTGGATGCAGCCCAGGCCCGGCCCTCGGACAACTGTGTC

301    TTCTCAGTGGAGTTGCAGCTGCCTCCCAAGGCTGCAGCCCTGGCTCACTTGGACAGGGGG
       ||||||||||||||||||||||||||||||||||||||||||||||||||||||||||||
301    TTCTCAGTGGAGTTGCAGCTGCCTCCCAAGGCTGCAGCCCTGGCTCACTTGGACAGGGGG
```

Supplementary Figure S3. Results of pAC-CAG-P2A-SSAO gene sequence comparison.

The bacterial solution of the positive clone pAC-CAG-P2A-SSAO expression vector was sent to Generay for sequencing. The sequencing results were compared with the original sequence of SSA0 (KEGG, 3569) by the software "DNAMAN". The results showed that the clone completely matched the original sequence of SSAO, and the homology reached 100%.

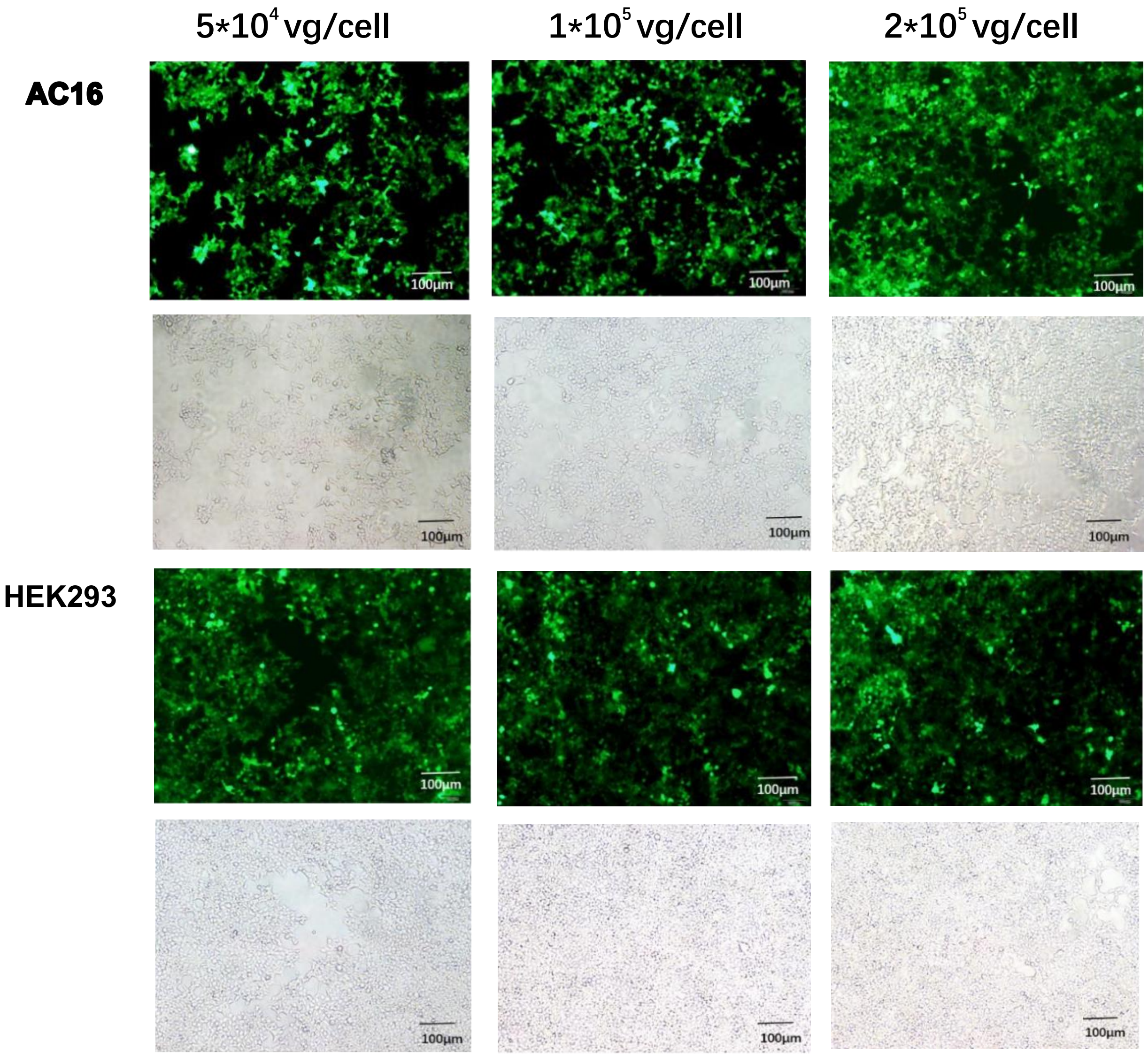

Supplementary Figure S4. pAC-CAG-P2A-EGFP plasmid was transfected into HEK293 cells and AC16 cells for 48h.

In this study, transfection efficiency was investigated through the constructed pAC-CAG-P2A-EGFP plasmid, because the pAC-CAG-P2A-EGFP plasmid contained green fluorescent protein reporter gene, which produced green fluorescence after excitation by blue band laser, and the intensity of fluorescence was positively correlated with transfection efficiency. The transfection efficiency was evaluated by combining cell state and fluorescence intensity. In summary, we selected the best transfected cells for 48H.

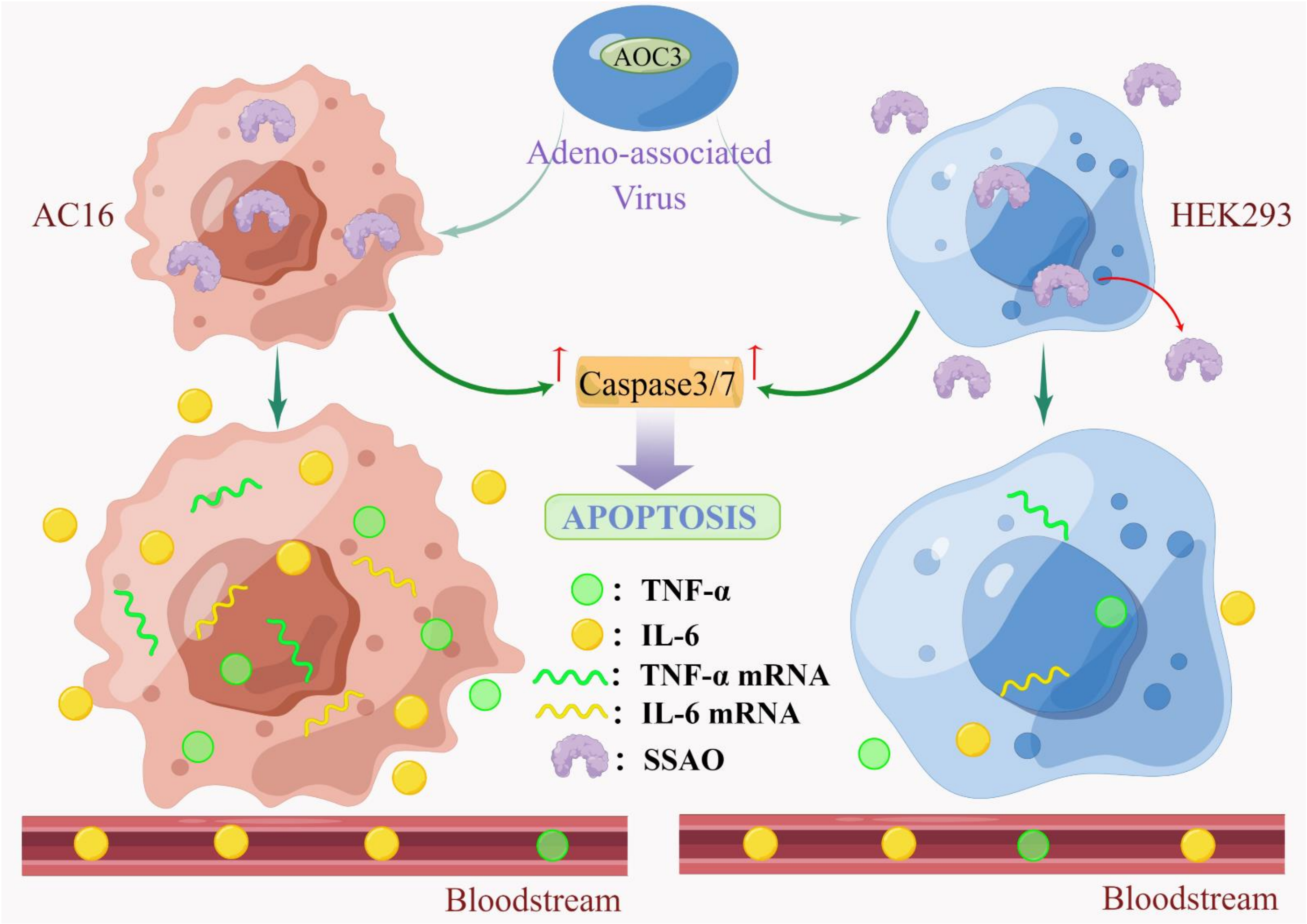

Supplementary Figure S5. The mechanism of SSAO overexpression caused different inflammatory responses in AC16 cells and HEK293 cells.
